# Slow-moving and far-travelled dense pyroclastic flows during the Peach Spring super-eruption

**DOI:** 10.1038/ncomms10890

**Published:** 2016-03-07

**Authors:** O. Roche, D. C. Buesch, G. A. Valentine

**Affiliations:** 1 Laboratoire Magmas et Volcans, Université Blaise Pascal-CNRS-IRD, OPGC, 6 Avenue Blaise Pascal, TSA 60026-CS 60026, F-63178 Aubière, France; 2 United States Geological Survey, 345 Middlefield Road, MS 973, Menlo Park, California 94025, USA; 3 Department of Geology and Center for Geohazards Studies, University at Buffalo, Buffalo, New York 14260, USA

## Abstract

Explosive volcanic super-eruptions of several hundred cubic kilometres or more generate long run-out pyroclastic density currents the dynamics of which are poorly understood and controversial. Deposits of one such event in the southwestern USA, the 18.8 Ma Peach Spring Tuff, were formed by pyroclastic flows that travelled >170 km from the eruptive centre and entrained blocks up to ∼70–90 cm diameter from the substrates along the flow paths. Here we combine these data with new experimental results to show that the flow's base had high-particle concentration and relatively modest speeds of ∼5–20 m s^−1^, fed by an eruption discharging magma at rates up to ∼10^7^–10^8^ m^3^ s^−1^ for a minimum of 2.5–10 h. We conclude that sustained high-eruption discharge and long-lived high-pore pressure in dense granular dispersion can be more important than large initial velocity and turbulent transport with dilute suspension in promoting long pyroclastic flow distance.

## Abstract (web summary)

Pyroclastic flows of the 18.8 Ma Peach Spring Tuff super-eruption travelled >170 km and entrained substrate blocks up to ∼1 m diameter. Here, the authors show that these flows had a dense base and speeds of ∼5-20 m s^−1^, fed by an eruption discharging magma at rates up to ∼10^7^-10^8^ m^3^ s^−1^ for at least 2.5-10 h.

## Graphical abstract

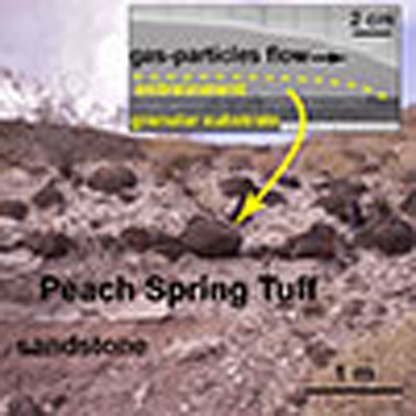

Explosive volcanic super-eruptions expel magma volumes of several hundred cubic kilometres or more and generate particle-gas flows called pyroclastic density currents^1^,^2^ (note we also use the term pyroclastic flow synonymously with pyroclastic density current, with no implications related to dynamics). There are many locations around the world where the deposits of these pumice-rich currents (ignimbrites) extend >100 km from their source vents^3^,^4^,^5^,^6^. These surprisingly long run-out distances raise fundamental questions about the flow velocity and propagation mechanism. Specifically, does long run out require high initial flow speeds and mass fluxes near the eruptive source? Also, are the density currents fully turbulent and dilute or is much of their mass carried in concentrated, basal granular dispersions? It is commonly assumed that the long run out of large-volume pyroclastic density currents requires high initial (proximal) flow speeds of 100–300 m s^−1^, which might result from collapse of explosive eruption columns from heights of a few kilometres^3^,^7^,^8^. There are different views on the physics that control transport and deposition once a pyroclastic current starts. One end-member mechanism is that the currents must be fast and highly turbulent, carrying pyroclastic particles mainly as a dilute (<1 vol.%) suspended load^9^. The other end-member mechanism has flows propagating as dense (>30–40 vol.%) granular dispersions (with an accompanying more dilute upper part), in which high pore gas pressure reduces internal friction^7^ and allows nearly inviscid flow while a deposit aggrades upward from the base^10^,^11^. Arguments for and against each of these mechanisms have been mainly qualitative with a few exceptions^9^, because of the difficulty in relating deposit characteristics to quantitative fluid dynamics theory. This debate is not just academic; an understanding of the mechanisms by which pyroclastic flows propagate is essential to accurately forecast related hazards at active volcanoes.

Recent works suggest that the size (that is, weight) of lithic blocks entrained from the substrate by the pyroclastic flows and preserved within the resulting ignimbrites can be used to test emplacement models and to estimate parent flow densities (related to particle concentration) and speeds. Experiments^12^ demonstrated that lithic block size could be used to estimate the speed of a dense flow whose deposition forms an ignimbrite unit. Theoretical considerations^13^ show that dilute turbulent currents with realistic speeds (generally <100 m s^−1^ outside the proximal area) are capable of entraining blocks with diameters up to ∼10 cm from subhorizontal substrates, while dense granular flows can entrain blocks of a few metres size at lower speeds.

The Peach Spring Tuff (PST, southwestern USA) is an example of an ignimbrite that contains large (>10 cm) substrate-derived lithic blocks. It was deposited from pyroclastic currents that travelled more than 170 km from their source during the super-eruption at the Silver Creek caldera ∼18.8 Myr ago^14^. The dense-rock equivalent volume of erupted material was >1,300 km^3^; at least half was deposited regionally by pyroclastic density currents^15^,^16^,^17^,^18^, and an estimated equal amount was trapped in the subsiding caldera^14^. The extra-caldera ignimbrite is poorly sorted and massive with an ash matrix that comprises 60–80% by volume of the deposit, based on field and thin section analysis^17^,^19^. The ignimbrite ranges from non-welded to densely welded, and vitric to crystallized (feldspar and cristobalite) as a result of hot emplacement, which suggests little mixing with ambient air during flow. It originally covered a broad area of ∼32,000 km^2^ over what is now western Arizona, southeastern California and southern Nevada. The local deposit thickness is 5–40 m at most sites, with local increase to ∼220 m in topographic depressions where the material accumulated, and the most distal deposits are >10-m thick^15^,^16^. The ignimbrite qualifies as a low aspect ratio deposit (a.r., ratio of average deposit thickness to the diameter of a circle that has the same area as that of the deposit), with a.r.∼10^−4^ and average thickness of ∼30 m comparable to similar deposits^4^,^5^,^7^,^20^. The parent pyroclastic flows propagated mainly over regional subhorizontal or gently sloping terrains that included alluvial fans with abundant loose clastic surface material, and varied local low to moderate relief including scoria cones and hills up to ∼200-m high. High mountains blocked the flow^15^,^16^,^19^,^21^. Post-depositional erosion and regional extension broke up the original sheet-like deposit so it now occurs in scattered outcrops as far as 240 km from the source caldera.

Here we combine field data on entrained lithic blocks with experimental results to show quantitatively, for the first time, that the basal portions of the parent flows of the PST super-eruption had high-particle concentration and relatively modest and uniform speed of ∼5–20 m s^−1^ even though the distance travelled exceeded 170 km. We report distances from the Silver Creek caldera that have been corrected for regional extension and strike-slip translation up to ∼80 km across three structural domains in California and up to ∼12 km across the Colorado Plateau Transition Zone (CPTZ; see Methods). The results further suggest the currents were fed by an eruption discharging magma at rates up to ∼10^7^–10^8^ m^3^ s^−1^ for a minimum of 2.5–10 h. These rates are 2–3 orders of magnitude larger than well-documented pre-historical or historical eruptions. We conclude that the long travel distances of such pyroclastic flows are related mainly to sustained high mass flux of material erupting from the volcano along with slow gas pore pressure diffusion within the flows due large amounts of ash, rather than from high initial flow velocities and/or transport via dilute turbulent suspension.

## Results

### The PST

**Figure 1:**
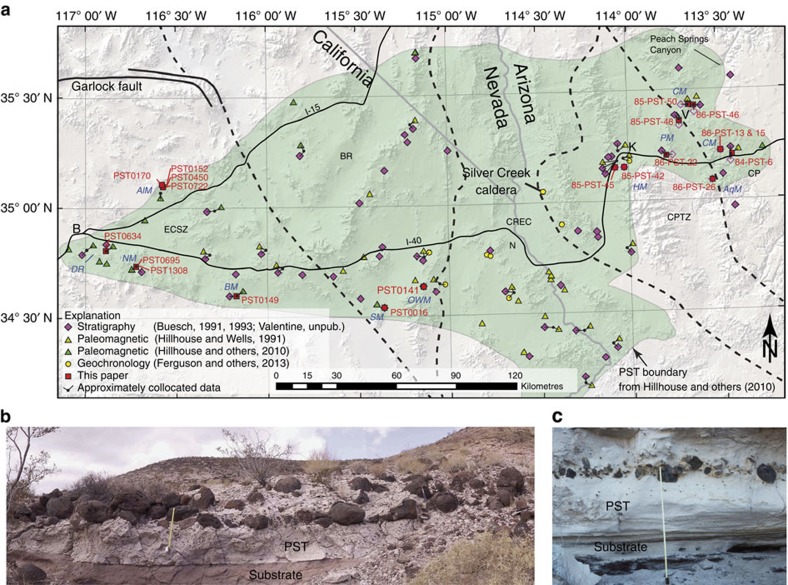
Map of the Peach Spring Tuff and locally derived lithic clasts. (**a**) The map shows locations with stratigraphic, paleomagnetic and geochronologic data used to correlate the Peach Spring Tuff (PST). Labelled are critical locations from this paper (Supplementary Table 1), Silver Creek caldera and structural-tectonic domains^14^,^18^,^35^. Cities—B, Barstow; K, Kingman; N, Needles; V, Valentine. Structural domains—ECSZ, Eastern California Shear Zone; BR, Basin and Range; CREC, Colorado River Extensional Corridor; CPTZ, Colorado Plateau Transition Zone; CP, Colorado Plateau. Mountains—AlM, Alvord Mountain; AqM, Aquarius Mountains; BM, Bullion Mountains; CM, Cottonwood Mountains; DR, Dagett Ridge; HM, Hualapai Mountains; NM, Newberry Mountains; OWM, Old Woman Mountains; PM, Peacock Mountains; SM, Ship Mountains. (**b**) Photograph at PST0695 in Kane Wash, Newberry Mountains, California (tape is 50 cm). The PST lies on a sandstone substrate. (**c**) Photograph at 85-PST-50 near town of Valentine, Arizona (tape is 1 m). The PST ignimbrite lies on ash layers that record initial phases of the PST eruption and covers fluvial sediments.

**Figure 2:**
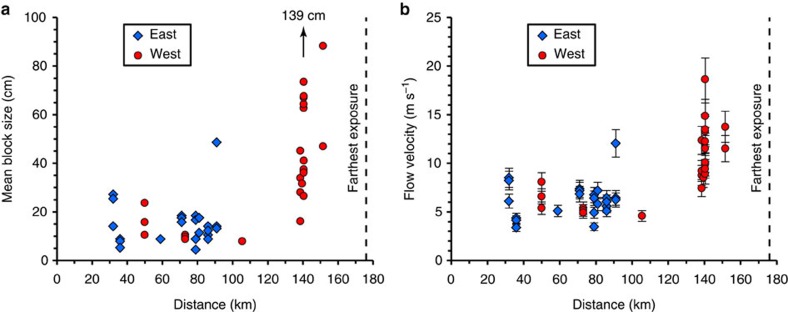
Characteristics of substrate-derived blocks in the Peach Spring Tuff. (**a**) Mean size of the largest blocks (the arrow indicates a large block at PST0695). (**b**) Corresponding velocity of the parent pyroclastic flows calculated from equation (1) as function of the corrected distance from the Silver Creek caldera. Error bars represent the range of velocities calculated.

**Figure 3:**
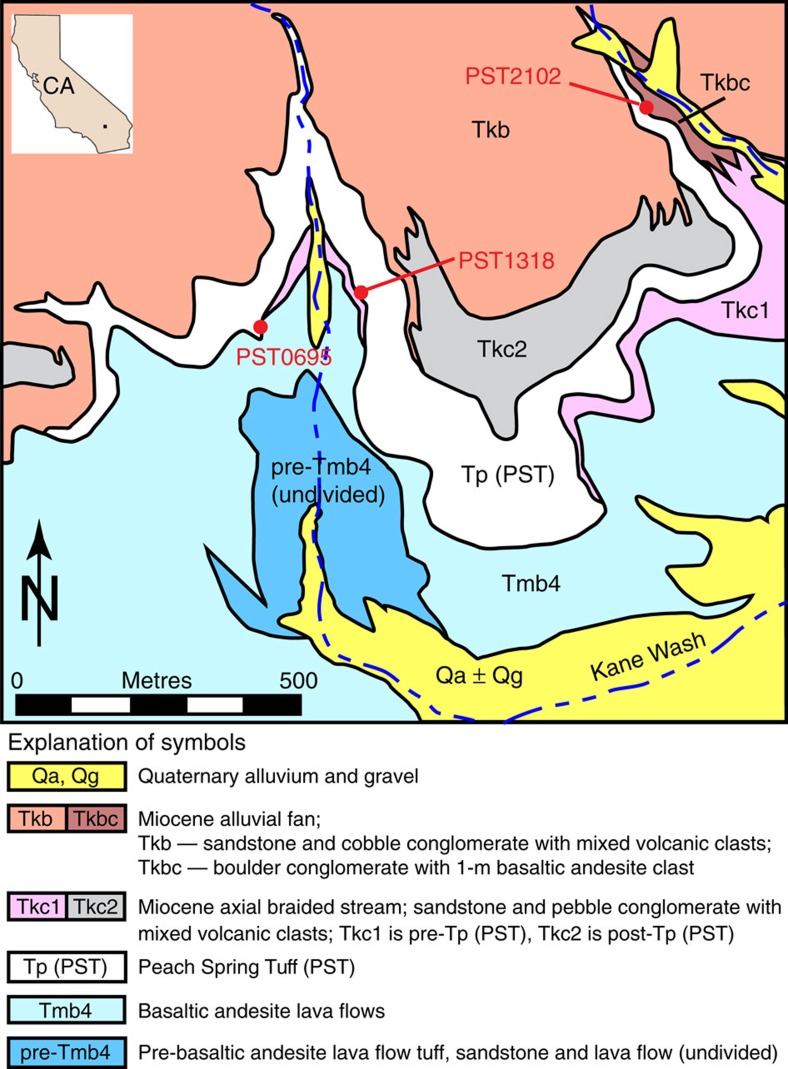
Geologic map of the Kane Wash area in the Newberry Mountains in California. It shows the westward-draining Kane Spring palleovalley (updated from unpublished map by Brett Cox, USGS). Locations of large substrate-derived lithic clasts at base of Peach Spring Tuff at PST0695 and PST1318, and a substrate conglomerate at PST2102 are indicated. Map unit Tkbc is the most likely source area of the clasts. Dashed line patterns represent streams.

The PST contains lithic blocks entrained from the local substrates by the pyroclastic currents and deposited within the ignimbrite^19^. We have identified 20 key outcrops at extension-corrected distances of ∼30–150 km to the east and west of the Silver Creek caldera (Fig. 1, Supplementary Table 1 and Figs 1, 2, 3). Closer outcrops are lacking due to post-eruptive faulting and burial in subsiding basins on either side of the caldera. Locally derived lithic block types correspond to rock types in the local substrates. To the west of the caldera these include various volcanic, granitoid and metamorphic rocks that were present essentially in and on alluvial fans and also on local highlands at the time of eruption^19^,^22^. East of the caldera, the lithics are mainly basaltic and granitic, corresponding to ignimbrite emplacement onto Precambrian granite locally covered by Cenozoic basaltic lavas and scoria and with broad fluvial channels containing those clast types^12^,^15^.

Substrate-derived blocks larger than 10 cm are found at almost all of the studied outcrops, with mean size up to 70–90 cm for blocks at sites ∼140–150 km (corrected) west of the vent with one outlier of 139 cm at ∼140 km (corrected) (Fig. 2). Entrainment of the typical 10 cm blocks from a subhorizontal substrate into a dilute, turbulent current would require flow speeds >100 m s^−1^ at heights of a few hundred metres [Fig f4](see Fig. 5a of ref. 13) that are maintained over many tens of kilometres of flow distance. This conclusion holds even if the maximum speed considered is at about one fifth the current height as assumed for more realistic velocity profiles typical of natural currents. The largest blocks, in particular, could not have been entrained by dilute pyroclastic currents because the required speed would have been >200–650 m s^−1^ (at one fifth the current height), which is unrealistic at this distance from the source^13^ (Supplementary Fig. 4 and Supplementary Discussion; compare, for example, maximum current speeds of ∼170 m s^−1^ at 4–6 km from vent for the lateral blast eruption at Mount St Helens, 1980 (ref. 23)).

**Figure 4 f4:**
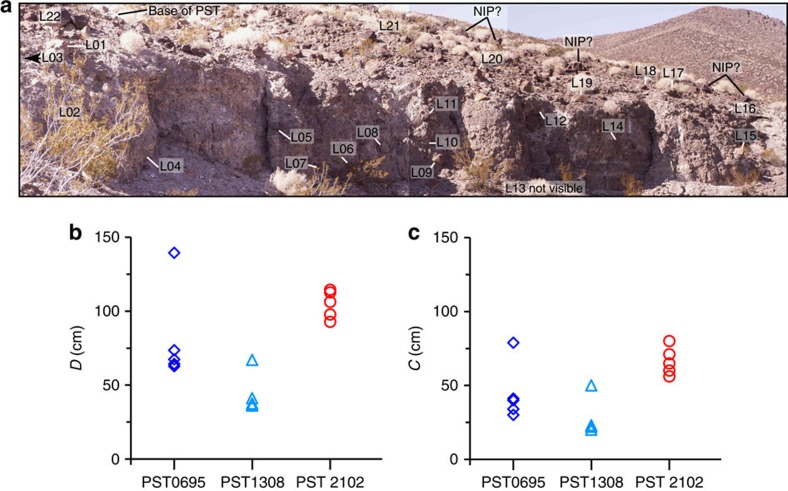
Size of the blocks in the Peach Spring Tuff and their source area at three locations in Kane Wash in California. (**a**) Photograph at location PST2102 of the large boulder conglomerate (Tkbc in Fig. 3). Blocks L01-22 were measured (for scale L22, top left, is 62 × 110 × 110 cm). Note the base of the Peach Spring Tuff (PST) on the top left is covered by <2 m of talus. Boulders up to 1.5 m diameter on the ground surface (possibly not in place, noted NIP?) are either from the pre-PST or post-PST conglomerate, and indicate the large size of clasts in these conglomerates. (**b**,**c**) Size of the five largest blocks in the Peach Spring Tuff at PST0695 and PST1308 (see Fig. 3), and at top of the pre-PST conglomerate at PST2102. (**b**) *D* is the equivalent diameter of the blocks. (**c**) *C* is the short length of the blocks.

The transport distance of the blocks by the pyroclastic currents is estimated to be up to several hundreds of metres, based on detailed field mapping from earlier studies^16^,^19^ and our recent field work (Fig. 3 and Supplementary Discussion). In the Kane Wash area, California, the northern flanks of the Kane Spring paleovalley reveal a conglomerate with subrounded basaltic-andesite boulders that are up to 1–1.2 m in diameter (identified in Fig. 3 by unit Tkbc) exposed just below the PST (Fig. 4a). This boulder conglomerate is the most likely source of the lithic clasts found in the PST about 650–800 m downstream to the west-southwest at locations PST0695 and PST1308 where the nature and shape of the blocks are similar to those in the conglomerate (Supplementary Figs 1 and 2). Field evidence suggests that the locally derived lithic clasts were incorporated into and redistributed within independently moving, relatively small pyroclastic flows or within a single, main pyroclastic flow to form lithic-rich horizons^19^. Our new field work at location PST0695, in particular, shows that the lowest 2 m of the PST contains a concentration of numerous large, subrounded basaltic-andesite lithic clasts of mean size up to >60–70 cm and whose bottoms are about 50 cm above the base of the PST (Fig. 1b, Supplementary Fig. 1 and Supplementary Table 1).

To address the entrainment mechanism of the substrate-derived lithic clasts found in the PST, and considering that the parent pyroclastic density currents could not be fully dilute turbulent mixtures (as stated above) and rather had a dense basal granular dispersion, we conducted a series of laboratory experiments on dense gas-particle flows propagating on a granular substrate.

### Experiments

**Figure 5:**
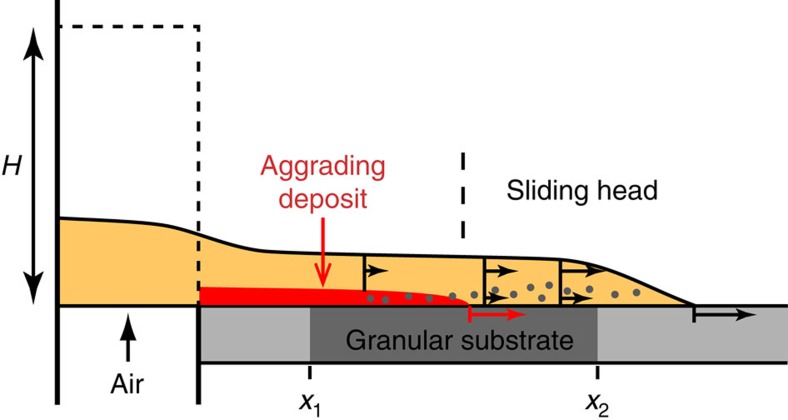
Schematic of the experimental device used in this study. The flow of fine (80 μm) particles, generated from a reservoir (dashed rectangle) by release of a fluidized granular column with high interstitial air pore pressure, entrains 1.6 mm diameter steel beads (dark grey dots) that form initially a granular substrate at distance between *x*_1_ and *x*_2_ (see Supplementary Table 2). The rigid substrate (light grey) before and beyond the granular substrate is either smooth or made rough by gluing a layer of glass beads of diameter of 0.7 or 1.5 mm. Distance of entrainment of substrate beads is not to scale. Horizontal arrows indicate relative velocities at given height above the substrate; the large arrows below the top of the substrate represent the front velocity of the flow (black) and of the advancing aggrading basal deposit (red).

We performed experiments on dense granular flows of fine (<80 μm) particles with high pore gas pressure propagating on a loose granular substrate of coarse (∼1.6 mm) beads inserted into a rigid substrate, as analogues to the concentrated basal parts of pyroclastic currents over local erodible substrates (Fig. 5 and Methods, see Supplementary Movies 1–6). Such flows have a fluid-like behaviour and propagate as (almost) inviscid mixtures until either pore pressure diffuses out or material supply is exhausted^10^. When propagating on a granular substrate, the sliding head of the flow generates both shear and a short-lived upward pore pressure gradient at the flow-substrate interface^12^. Shear promotes extraction of the substrate particles, which are first dragged slowly over a distance of a few bead diameters just above the top of the substrate before being uplifted at a given distance behind the flow front (or leading edge) because of the pressure gradient. Laboratory experiments^11^,^12^ demonstrate that the pressure gradient initially increases with time after passage of the flow front, and the onset of uplift occurs at a critical upward pressure gradient whose associated uplift force counterbalances the weight of individual beads (see Fig. 4 of ref. 12). This shows that the pressure gradient, which is proportional to the square of the flow front velocity, is the main cause of the onset of uplift of the substrate beads dragged at the top of the substrate. Experiments involving beads with different densities but the same size (that is, different weight) reveal different critical pressure gradients and further confirm that the model of ref. 12 we adopt hereafter is robust with respect to the clear relationship between the onset of particle uplift and the square of the front velocity. Other mechanisms, including those similar to that in single-phase fluid flows (for example, Basset and Magnus forces)^24^, as well as kinetic sieving known for dry granular flows^25^ might occur but appear to be minor influences in the experiments in promoting onset of uplift. Reference^12^ points out that though particle uplift by granular flows shares similarities with that of single-phase fluid flows, the shear stress and vertical forces over the substrate particles are of different natures. Nevertheless, kinematic sieving and squeeze expulsion caused by particle interactions can contribute to controlling the rise height of the beads once the pore pressure gradient has caused onset of uplift. As discussed by ref. 13, however, large beads whose density is larger than the bulk density of the fluidized mixture of fines with high pore fluid pressure should ultimately sink because of buoyancy effects, which actually occurred in our experiments as described below.

**Figure 6:**
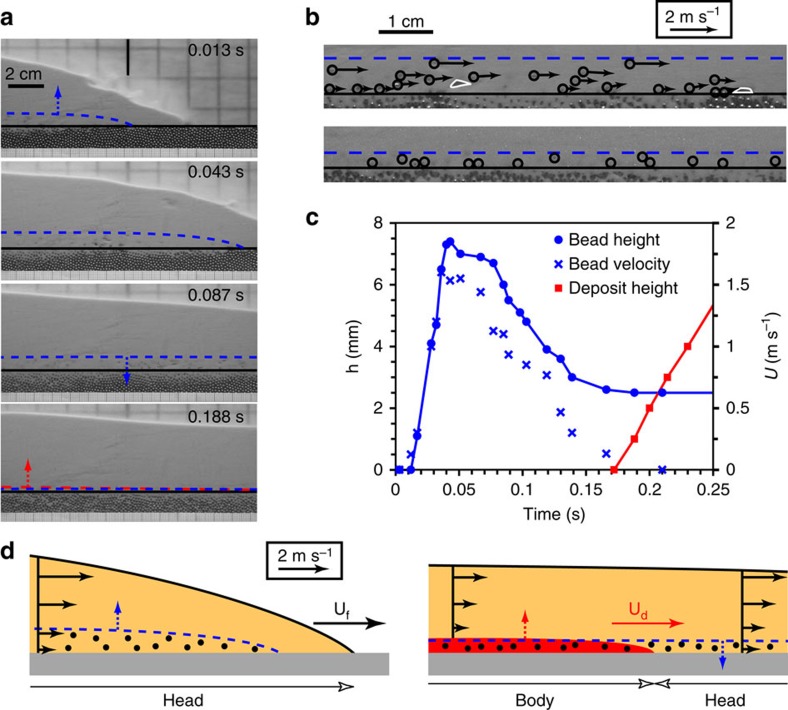
Laboratory experiment of air-particle flow on a granular substrate. (**a**) Snapshots from high-speed videos at sequential times after passage of the flow front at middle of images (vertical black line) in experiment C3 (repeated six times, Supplementary Table 2). The horizontal black line shows the top of the substrate of steel beads. Blue and red dashed lines indicate the upper surface of the zone of the entrained substrate beads and of the basal deposit, respectively, and arrows show relative motion. (**b**) Detailed views of (top) the flow at 0.048 s and (bottom) the final deposit (note white flow fines penetrating into the substrate interstices). Arrows indicate the direction and velocity of the entrained beads (circled), and white contours delimit air bubbles. Note that beads have either ascending (uplift) or descending (settling) trajectories. (**c**) Height of the upper surface of the zone of the entrained beads and of the basal deposit above the substrate (*h*) and velocity of the uppermost beads (*U*) as a function of time. The particle velocity increases upward, similar to the local internal flow velocity. (**d**) Schematic successive views showing (left) beads (black) entrained from the substrate (grey) by the sliding flow head, and (right) the advancing aggrading basal deposit (red) that freezes beads entrained downstream and that finally settle back towards the substrate. Horizontal arrows represent the internal flow and beads' velocity as well as the flow front (*U*_f_) and deposit advancing front (*U*_d_) velocities ∼2.5 m s^−1^, higher than the maximum entrained beads velocity (∼1.6 m s^−1^ in **c**).

Our experiments involved a substrate of steel beads of diameter ∼1.6 mm (Supplementary Table 2). They were carried out at flow front velocities >0.97 m s^−1^ that caused a pressure difference >82 Pa required for uplift of the steel beads^12^. They show how during flow propagation, substrate particles are entrained within a basal zone whose upper surface migrates first rapidly upwards to a height of ∼5–8 mm, as beads are uplifted, and then slowly downwards, as beads settle (Fig. 6, Supplementary Fig. 5). High-speed videos reveal that the particle velocity increases upward, similar to the local internal flow velocity, and that at any given time some beads have ascending (uplift) trajectories while others have descending (settling) trajectories. The transport distance of uplifted beads cannot be determined accurately since it exceeds the field of observation and entrained particles are hidden intermittently by the matrix of fines, but it can be relatively large (up to ∼1–1.5 m) once the flow propagates onto a rigid substrate downstream. Another important observation is that entrained particles are overtaken from below by the advancing front of the aggrading deposit that forms at flow base (Fig. 6d). This front begins a few centimetres behind the flow front and advances at a similar speed. Therefore, substrate-derived beads are deposited downstream near the base of deposits that form either on granular or rigid substrates. The final resting height of a given entrained particle is determined by a competition between the particle's uplift and descent history, and the upward-advancing aggradation surface at a given location.

### Pyroclastic flow speeds and eruption rates

According to experimental findings and theory^12^,^13^, a flow of front velocity *U*_f_ entrains blocks whose short (subvertical) length is up to a critical value *C*. Using *C* based on our field observations (Supplementary Table 1), we can calculate the front velocity from


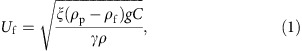


where *ξ* is a shape factor (equal to 2/3 for an ellipsoid and 1 for a parallelepiped^13^), *ρ*_p_ is the block density, *ρ*_f_∼1 kg m^−3^ is the gas density, *g* is the gravitational acceleration, *γ*≈0.06 is an empirical factor^12^ and *ρ*=875–1,400 kg m^−3^ is the bulk flow density^12^,^13^ (see Methods). From equation (1) (with *ξ*=1), the largest blocks at the PST key outcrops give flow speeds of ∼5–20 m s^−1^ across large flow distances with different substrates (Fig. 2b). The relative uniformity of the speed estimates suggests that the 5–20 m s^−1^ range is realistic. Furthermore, the fact that many of the largest blocks in the ignimbrite are just smaller than the largest blocks remaining on the substrate in their source areas shows that the PST parent flows sampled blocks up to a critical size and that our estimates are not simply minimum speeds related to the lack of sufficiently large blocks in the original substrates (Fig. 4 and Supplementary Discussion). For instance, in the Kane Wash area (Fig. 3), the five largest substrate-derived blocks at sites PST0695 and PST1308 in the PST are just smaller (with the exception of the outlier 80 × 120 × 150 cm at PST0695) than the blocks located at top of the pre-PST conglomerate unit (PST2102) from which the boulders are inferred to have been entrained (Fig. 4), which suggests that our calculated flow velocity up to ∼15–20 m s^−1^ at these sites is realistic.

Flow speeds of 5–20 m s^−1^ and the run-out distance of ∼170 km correspond to a minimum flow duration of ∼2.5–10 h, which does not account for the time needed to aggrade the final deposit or to stack flow units if the PST emplacement involved pulses (we note, however, that field evidence at many PST sites suggest one single flow unit^15^,^19^). These durations are reasonable if internal gas pore pressure, which greatly reduces internal friction in the currents, is long-lived and if a sufficient pressure head provided by the drop height from sustained fountaining at the vent(s), enhanced by gentle slopes away from the source area, is maintained along the current's path^10^,^20^. The pore pressure diffusion timescale is estimated from


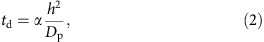


where *α*∼1.7, *h* is the flow thickness and *D*_p_ is the pressure diffusion coefficient^11^,^26^. The 2.5–10 h flow durations are compatible with pore pressure diffusion timescales in non-expanded ash-rich pyroclastic mixtures with *D*_p_∼10^−1^–10^−2^ m^2^ s^−1^ (that is, hydraulic permeability ∼10^−13^–10^−12^ m^2^, refs 11, 27) and thicknesses of ∼5–20 m, or in even thinner flows of the same but moderately expanded material^11^. Such flow thicknesses are smaller than the thickness of most PST deposits, which are formed by aggradation of the flow(s). Assuming a total eruption volume of ∼1,300 km^3^, a magma density of 2,500 kg m^−3^ and the minimum flow duration above leads to volume and mass eruption rates as high as ∼3.8 × 10^7^–1.5 × 10^8^ m^3^ s^−1^ and ∼9.6 × 10^10^–3.8 × 10^11^ kg s^−1^, respectively.

## Discussion

The maximum eruption rates we report show that the intensity of the PST eruption relative to eruptions within human experience was enormous. Compared with historical well-documented eruptions, the rates are equivalent to ∼80–300 times the flux of the climactic phase of the 1991 Mount Pinatubo eruption^28^ (∼12 × 10^8^ kg s^−1^) or ∼600–2,500 times the maximum discharge rate during the Plinian phase of the AD 79 eruption at Vesuvius^29^ (∼1.5 × 10^8^ kg s^−1^). Unlike Pinatubo and Vesuvius, the PST eruption must have involved many simultaneously active vents as the Silver Creek caldera foundered and collapsed. Compared with other caldera-forming eruptions, whose intensities were calculated by methods different to the one we present here, our maximum rates are in most cases 1–3 orders of magnitude higher. The strongest differences are found for eruptions of relatively small (<60 km^3^) volume such as the Kos Plateau Tuff^30^, ∼161 ka (∼1.8 × 10^6^−3.6 × 10^6^ m^3^ s^−1^), Tambora^31^, 1,815 (5 × 10^8^ kg s^−1^) and Novarupata^32^, 1,912 (2 × 10^7^−2 × 10^8^ kg s^−1^), which could be related to calderas being smaller than the Silver Creek caldera and with fewer active vents. Our calculated eruption rates, however, encompass that of the ∼1.8 ka Taupo eruption (∼30 km^3^, ∼5 × 10^7^ m^3^ s^−1^) given by ref. 9 from modelling of the parent pyroclastic density currents based on assumed turbulent and fully dilute (solid concentration of 0.3% vol.) mixtures of typical speed of 200 m s^−1^, which is in sharp contrast with the emplacement mechanism we propose for the PST. On the other hand, the values we report are only ∼10–50 times higher than that of the ∼75 ka Toba super-eruption^31^ (7.1 × 10^9^ kg s^−1^) with a volume ∼2,840 km^3^ that is of the same order of magnitude as that of the PST eruption.

This study shows that substrate-derived lithic blocks in ignimbrites can be used to constrain the emplacement mechanisms of the parent flows. Our new quantitative data on the PST indicate that some extremely mobile, large-volume pyroclastic currents that form low aspect ratio ignimbrites can have dense basal portions^7^ and we show that these flows can have relatively modest speeds. These pyroclastic currents likely also have an upper more dilute part with a thickness of up to a few hundreds of metres and that may form deposits on topographic highs of similar heights. Also, we do not rule out high flow velocities typically >100 m s^−1^ in proximal areas (<30 km according to Fig. 2) before the mixture decelerates and possibly deflates. Hence, high velocity and/or fully dilute turbulent currents^9^ are not required to form all low aspect ratio ignimbrites (as previously pointed out in ref. 20), raising questions about the general applicability of dilute, turbulent transport models of formation of such deposits, especially those where welding indicates hot emplacement and little mixing with air during flow. In fact, massive incorporation of ambient air into a hot and fast turbulent current rapidly reduces the mixture density, which causes lift-off and modest flow run-out distances^23^,^33^ (note that a dilute current can have long travel distances if it is fed by a dense underflow as we consider here). Instead, we conclude that the main factors in driving some currents are sustained extremely high discharge rates, which occur preferentially in caldera-forming eruptions, and abundant ash that reduces permeability of the dense basal granular dispersion and promotes long-lived gas pore pressure.

## Methods

### Correction of distances for the PST outcrops

Relative to the Silver Creek caldera (measured from sample CAF-2-21456 in Table 1 of ref. 14), the PST occurs about 110 km east the caldera in Peach Springs Canyon (the type section), and about 240 km west of the caldera in a 3.5-m-thick section ∼11 km southwest of Barstow, California^16^,^18^ (Fig. 1). The PST occurs in five structural domains: (1) the CP, (2) the CPTZ, (3) the Colorado River Extensional Corridor (CREC), (4) the Basin and Range (BR), and (5) the Eastern California Shear Zone (ECSZ) (Fig. 1). Structural extension or translation in the CPTZ, CREC and ECSZ has occurred after deposition of the PST, and there has been minimal extension or translation in the BR, and none in the CP.

Post-PST faulting contributed to the present-day measured distances from exposed PST relative to the caldera, and the amount of east–west extension and translation has been estimated to provide a minimum travel distance for the parent pyroclastic flow (Supplementary Table 1). Extension and translation are different types of relative separation along faults, and the east–west component of separation has been calculated. To the east of the caldera in the CPTZ, there might have been 5–12 km of post-PST extension (Supplementary Table 1), although no detailed studies of extension in this area have been done. Reference 34^34^ estimated that about 60 km of extension was the best fit for reconstructing anisotropy of magnetic susceptibility in the PST, and that 20% of the extension occurred in the CPTZ.

To the west of the caldera, from 26 to 50 km of post-PST extension occurred across the CREC with 18–34 km in a westward direction^14^,^35^, and a complicated and poorly constrained amount of left-slip on east-striking faults and right-slip on northwest-striking faults within the ECSZ^18^ might have added 30–50 km of westward distance between some locations and the caldera (Supplementary Table 1). In the ECSZ, some faults have strike-slip separation that results in an east–west distortion of the area. However, some faults with vertical axis rotation can have strike-slip separation that, depending on the block rotation geometry, can (or does not) contribute appreciably to an east–west distortion of the area. These possible vertical axis rotations were not specifically calculated, and the east–west apparent separation was calculated based on the values depicted in ref. 18. This simplification might result in larger amounts of apparent east–west translation than with other reconstructions, but these larger values result in shorter apparent travel distances of the PST parent pyroclastic flow. For each PST location in the ECSZ, in addition to the extension in the CREC, the amount of separation on specific faults between the caldera and the location were calculated. For example, the westernmost PST exposure is near Barstow and this is 240 km west of the caldera (Fig. 1); however, with the correction of the post-depositional extension-translation the flow distance was about 170 km.

### Block size and corresponding flow velocities

We have identified 20 key outcrops at extension-corrected distances of ∼30–150 km to the east and west of the Silver Creek caldera (Supplementary Table 1). The size of the substrate-derived blocks in the PST was measured in the field in most cases or from photographs with a well-defined scale when blocks were not accessible. Long (*A*), medium (*B*) and short (*C*) lengths were measured, and the mean block size was calculated assuming an equal-volume sphere of diameter *D*=^3^√(6*ABC*/*π*) (see Supplementary Table 1). For a few blocks, the medium length *B* could not be measured and it was calculated from the size data of other blocks for which *B*/*A*=0.64±0.12.

The velocities of the pyroclastic flows were calculated from equation (1) according to the method of ref. 12, which was validated by field data of the 1980 eruptions at Mount St Helens. Blocks were considered as ideal parallelepipeds (shape factor *ξ*=1) and their density was estimated from their petrographic type. Following ref. 12 a range of bulk flow densities was assumed, with a maximum value *ρ*∼1,400 kg m^−3^, equal to that of most non-welded PST deposits we measured for 19 stratigraphic sections and a minimum value *ρ*∼875 kg m^−3^ corresponding to a maximum expansion of ∼60% as shown in laboratory experiments on fluidized ash-rich pyroclastic mixtures^27^,^36^.

### Laboratory experiments

Experiments were conducted in the dam-break configuration, which consisted of the rapid release of a granular material from a reservoir that generated a gravitational flow in a 3-m-long channel (Fig. 5). They were filmed with a high-speed video camera at rates of 500–1,000 frames per s, and images were processed to analyse the detailed flow kinematics and to track the motion of the particles. In most experiments, the channel base consisted of a 3-cm-thick layer of spherical steel beads of diameter of 1.6 mm inserted into a rigid smooth or rough substrate at a given distance from the reservoir (Supplementary Table 2). Complementary experiments were carried out with glass beads of diameter of 1.5 mm beads but the entrainment mechanisms were not analysed in detail. The granular layer was built by leveling poured beads using a vertical plate translated horizontally along the channel so that the respective tops of the granular layer and of the rigid substrate were at the same horizontal level. The flow particles were fine glass beads of diameter of 80 μm, which permitted us to fulfil scaling requirements regarding the natural system^11^. Initial interstitial pore fluid pressure was generated by injecting air through a porous plate at the base of the granular column in the reservoir. At mean air flow velocity of 8 mm s^−1^, the pore pressure was high enough to counterbalance the particles' weight and to cause negligible interparticle frictional forces. The air-particle flow that was created as the sliding gate of the reservoir was released defluidized slowly during propagation through pore pressure diffusion owing to the low material permeability ∼10^−11^ m^2^ (ref. 11). The flow propagated at front velocity ∼√(2*gH*), where *g* was the gravitational acceleration and *H* was the initial granular column height^10^. The flow structure consisted of a sliding head (that is, non-zero basal velocity) and of an aggrading basal deposit whose front advanced at a speed similar to that of the flow front (Fig. 5). Shear at base of the sliding head caused entrainment of the substrate particles, which were uplifted because of an upward pore pressure gradient and transported downstream according to the complex internal flow velocity pattern. In fact, the flow velocity was maximal at the front (that is, leading edge), and it decreased backwards at a given level above the substrate while it increased upwards at a given distance behind the flow front (Figs 5, 6). In consequence, when the entrained particles finally settled towards the flow base they were overtaken by the (faster) advancing front of the basal deposit, which froze the particles above the top of the granular or rigid substrate depending on the distance from the reservoir.

## Additional information

**How to cite this article:** Roche, O. *et al.* Slow-moving and far-travelled dense pyroclastic flows during the Peach Spring super-eruption. *Nat. Commun.* 7:10890 doi: 10.1038/ncomms10890 (2016).

## Supplementary Material

Supplementary Information Supplementary Figures 1-5, Supplementary Tables 1-2, Supplementary Discussion and Supplementary References.

Supplementary Movie 1 Experiment C2 - Air-particle flow on a 3 cm-thick substrate of 1.6 mm diameter steel beads. Numbers indicate distance from reservoir gate to the edges of the field of view. Movie speed is 40 times less the actual speed.

Supplementary Movie 2 Experiment C3 - Air-particle flow on a substrate of 1.6 mm diameter steel beads. Distance from reservoir gate to the edges of the field of view is 73-87 cm. Graph paper for scale (fine lines are spaced at 1 mm, coarse lines at 1 cm). Movie speed is 40 times less the actual speed.

Supplementary Movie 3 Experiment C5 - Air-particle flow on a substrate of 1.6 mm diameter steel beads. Distance from reservoir gate to the edges of the field of view is 64-76 cm. Graph paper for scale (fine lines are spaced at 1 mm, coarse lines at 1 cm). Movie speed is 20 times less the actual speed.

Supplementary Movie 4 Experiment L4 - Air-particle flow at transition between a substrate of 1.6 mm diameter steel beads and a smooth rigid substrate (60 cm from reservoir gate). Graph paper for scale (fine lines are spaced at 1 mm, coarse lines at 1 cm). Movie speed is 40 times less the actual speed.

Supplementary Movie 5 Experiment L6 - Air-particle flow at transition between a substrate of 1.6 mm diameter steel beads and a 1.5 mm-rough rigid substrate (60 cm from reservoir gate). Graph paper for scale (fine lines are spaced at 1 mm, coarse lines at 1 cm). Movie speed is 40 times less the actual speed.

Supplementary Movie 6 Experiment L7 - Air-particle flow at transition between a substrate of 1.5 mm diameter glass beads and a 0.7 mm-rough rigid substrate (60 cm from reservoir gate). Graph paper for scale (fine lines are spaced at 1 mm, coarse lines at 1 cm). Movie speed is 40 times less the actual speed.
