# Supplementary material for: Slow-moving and far-travelled dense pyroclastic flows during the Peach Spring super-eruption
Source: Nat Commun. 2016 Mar 7;7:10890. doi: 10.1038/ncomms10890 (PMC4786679; doi:10.1038/ncomms10890)
Supplement: Supplementary Information — Supplementary Figures 1-5, Supplementary Tables 1-2, Supplementary Discussion and Supplementary References. [file ncomms10890-s1.pdf]

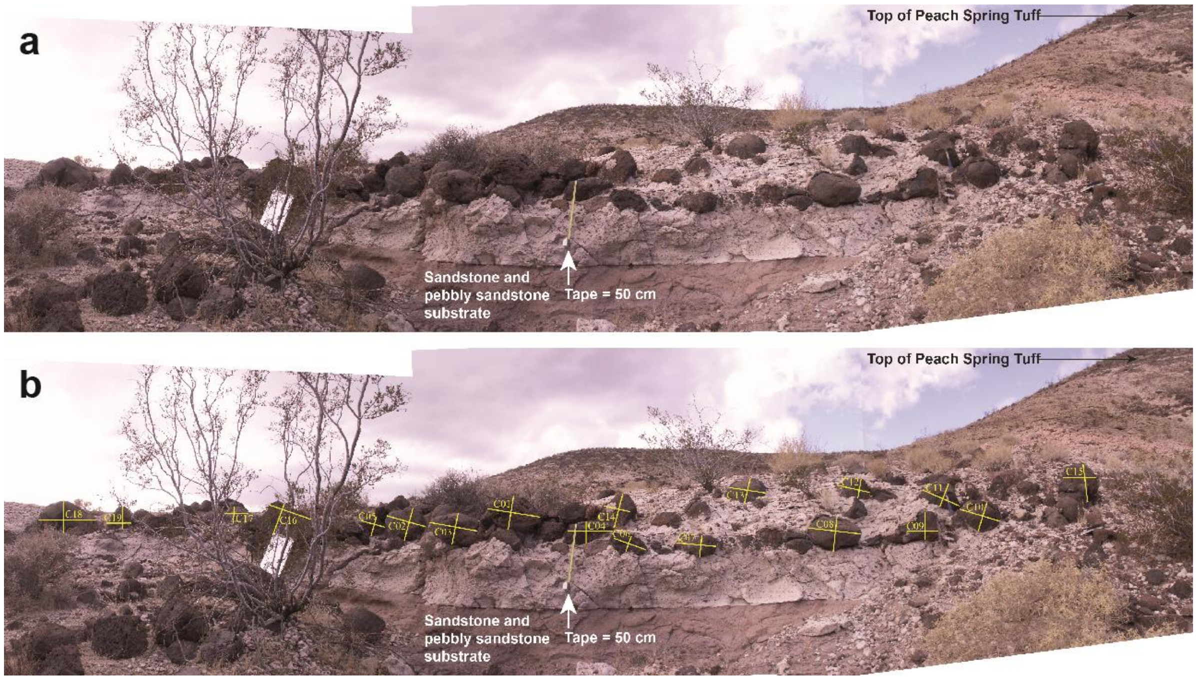

Supplementary Figure 1. Photograph assemblage of large lithic clasts near base of the Peach Spring Tuff at location PST0695, Kane Wash area, Newberry Mountains, California. **a,** View direction is to the west-southwest, approximately along direction of flow in the main, more ash-rich pyroclastic flow. The base of the ignimbrite is planar; however, in the panorama there appears to be a slight curvature due to image distortion. For scale, the tape is 50 cm, the hammer is 28 cm, and the map case is 46.5 cm long. **b,** Clast number with short and long (or medium) lengths of clasts showing partially developed imbrication from north (right) to south (left).

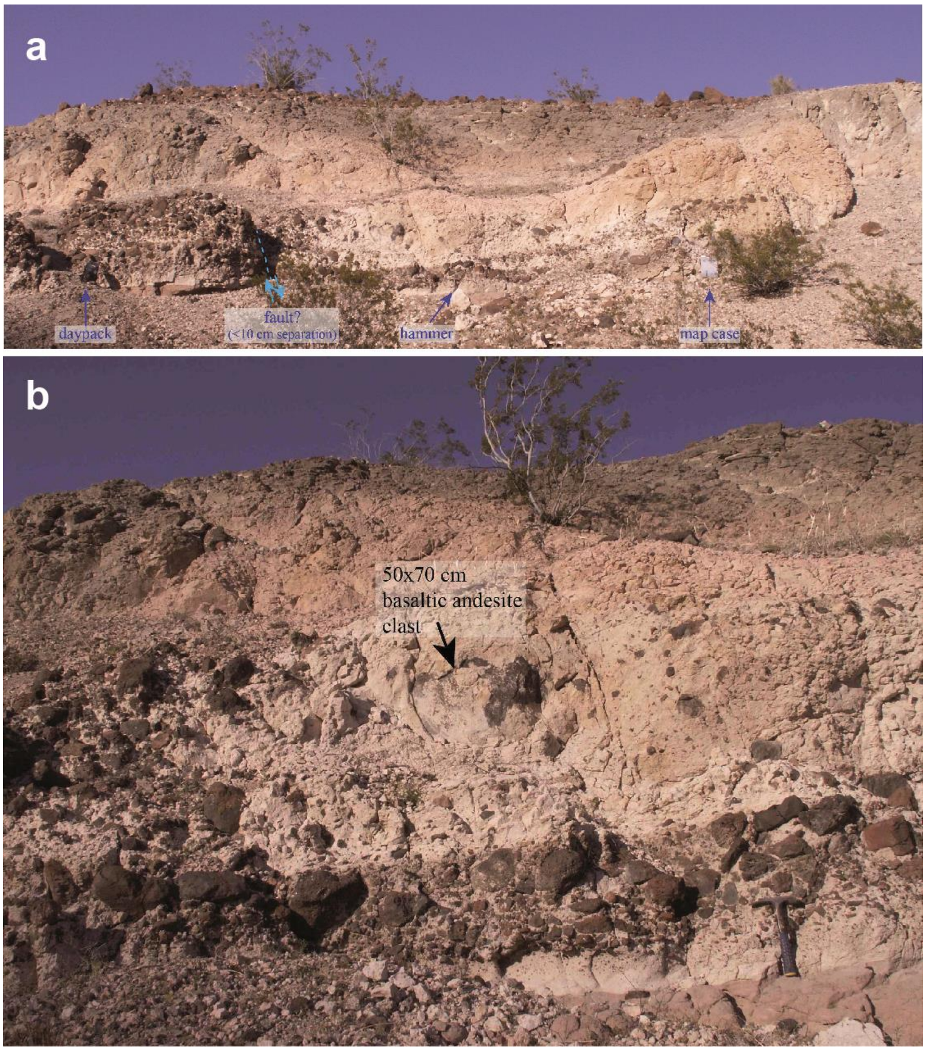

Supplementary Figure 2. Photograph at location PST1308 of large lithic clasts near base of the Peach Spring Tuff, Kane Wash area, Newberry Mountains, California. **a,** View direction is to the east-northeast, approximately opposite the direction of flow of the main, more ash-rich pyroclastic flow. Lithic-rich deposits thicken and thin in a complex interstratification. For scale, the daypack is 30 cm, the hammer is 28 cm, and the map case is 46.5 cm long. **b,** Detail of largest, basaltic andesite, lithic clast.

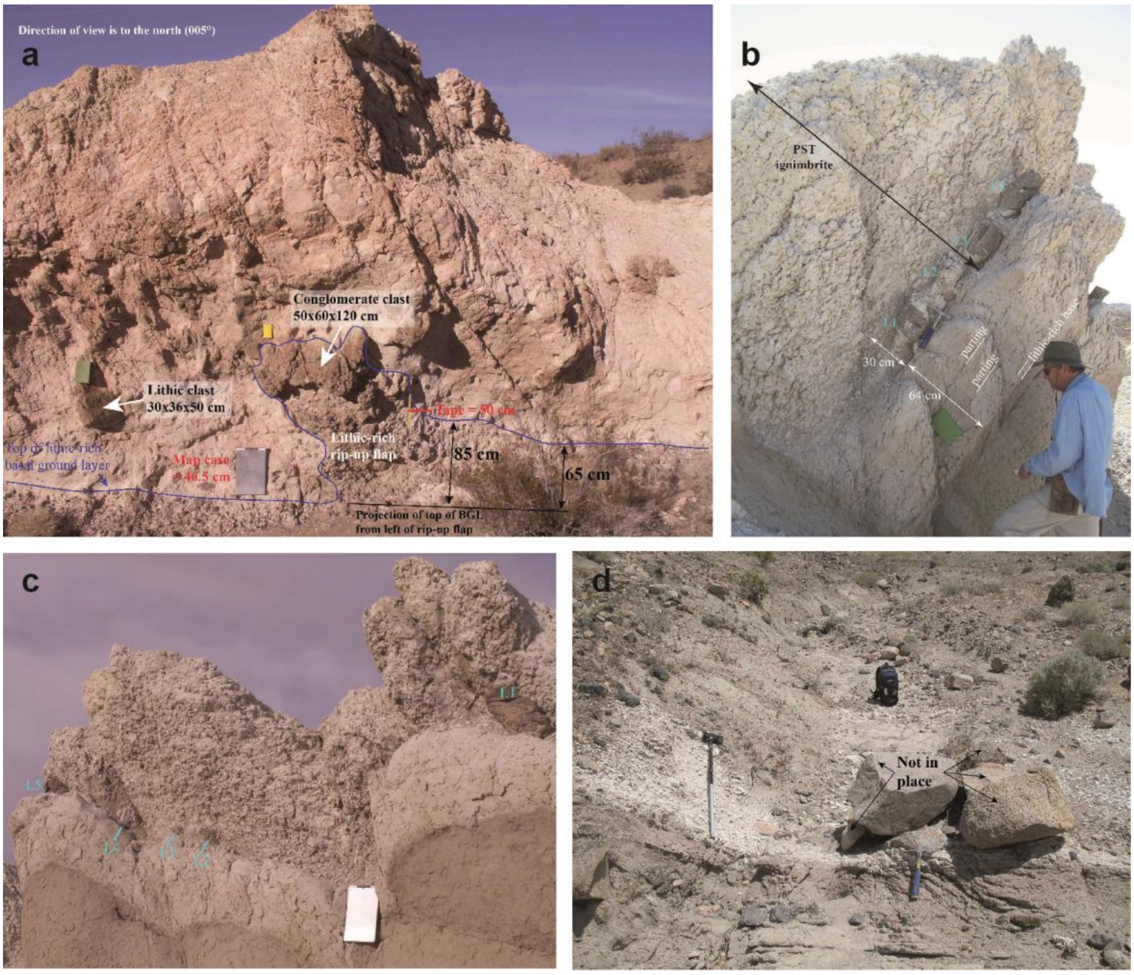

Supplementary Figure 3. a, Photograph at location PST0634 is of a 50-cm long isolated lithic clast, 120-cm long conglomeratic clast, and lithic-rich rip-up flap near the base of the PST, West Gem, eastern Daggett Ridge, California. Tape is 50 cm, map case is 46.5 cm, and green notebook is 20.3 cm long. **b,** Photograph at location AM0450 is of the lower part of the 3.3-m thick PST on the east limb of the Spanish Canyon anticline, Alvord Mountain area, California. The PST includes the basal ground layer (white annotations) and four, large, granitoid lithic clasts (L1-4) above it. **c,** Photograph at location AM0722 is of the lower part of the 3.3-m thick PST on the east limb of the Spanish Canyon anticline, Alvord Mountain area, California. The PST includes the basal ground layer and four, large, granitoid lithic clasts (L1-4) above it. Clast L5 is the upper end of clast L4 in **b**. **d,** Photograph at location AM0170 is of the 1.1-m thick PST on the east limb of the Spanish Canyon anticline, Alvord Mountain area, California. Jacob staff is 1 m, and is on the PST. The granitoid clast next to the hammer head is 17x39 cm, and the base of the clast is 8 cm above the base of the 15-cm thick basal ground layer.

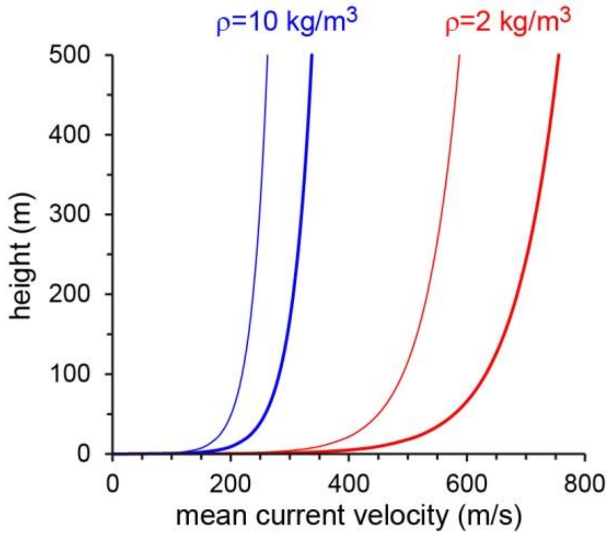

Supplementary Figure 4. Theoretical velocity of dilute turbulent pyroclastic currents required to entrain the largest PST substrate-derived blocks (80 cm), as a function of height above the substrate. Results are presented for lower and upper limit values of the current bulk density (ρ) and for Shield number values of 0.052 (thin lines) and 0.086 (thick lines).

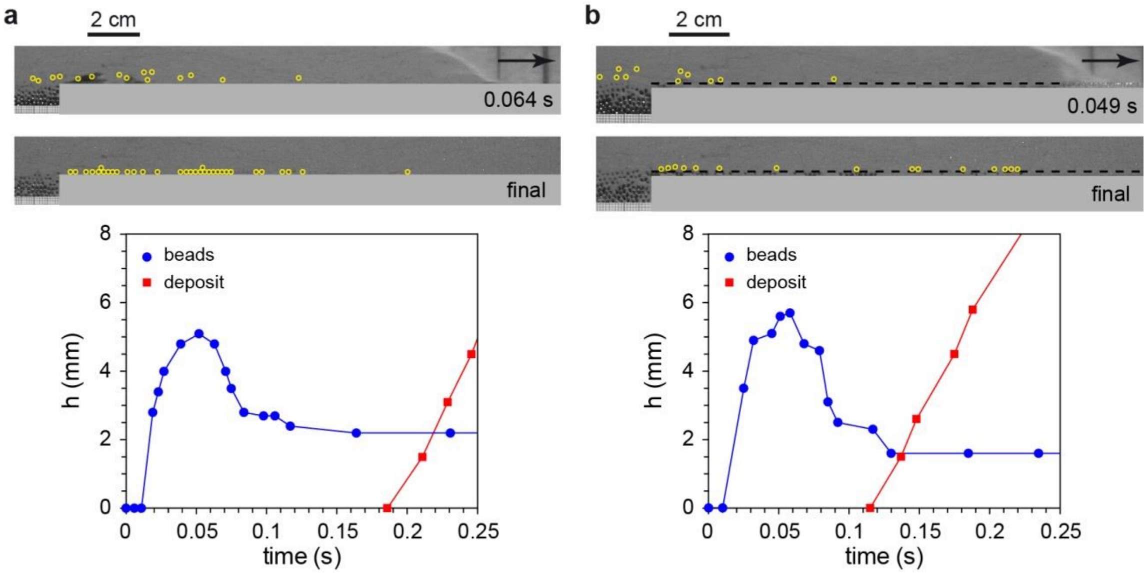

Supplementary Figure 5. Laboratory experiments of air-particle flow propagating on a granular substrate of 1.6 mm diameter steel beads and then on a: **a,** smooth (experiment L4, Supplementary Table 2) or **b,** rough (experiment L6, Supplementary Table 2) rigid substrate (grey). In the photographs, at given time after passage of the flow front at the granular-rigid substrate transition or showing the final deposit, the black horizontal arrows indicate the flow direction and yellow circles represent entrained substrate beads. Sampling of the deposit revealed that beads derived from the granular substrate were found downstream on the rigid substrate at distance up to ~1-1.5 m from the granular-rigid substrate transition. In **b** the horizontal dashed lines represent the top of the rigid rough substrate (glued 1.5 mm diameter glass beads). The graphs show the height of the upper surface of the zone of the entrained beads and of the basal deposit above the substrate (h) as a function of time (compare with Fig. 6 in paper); time zero corresponds to arrival of the flow front at the granular-rigid substrate transition.

Supplementary Table 1. Peach Spring Tuff (PST) outcrops with substrate-derived clasts larger than 5-10 cm.

Location	Latitude Longitude	Distance from caldera (real, corrected [a]) (km)	PST thickness (m)	Block type and origin	Block density (kg/m^3)	Block height above PST base (cm)	Size of largest blocks (cm)[b]	Flow velocity range (m/s)[c]
Eastern sector								
84-PST-6 Cottonwood Mtns, AZ	35°11’58.23’’N 113°25’47.62’’W	97, 91±6	15	basalt from fluvial sediments	3000	45	13, 14, 48 (8, 8, 29)	6.2-12.0
85-PST-42 Hualapai Mtns, AZ	35°9’8.05’’N 114°1’39.71’’W	42, 36±6	35	basaltic scoria from cone	2200	50	5, 8, 9 (3, 5, 5)	3.4-4.3
85-PST-45 Hualapai Mtns, AZ	35°9’8.18’’N 114°4’41.31’’W	38, 32±6	45	granite from topographic high	2700	50	14, 25, 27 (8, 15, 16)	6.1-8.4
85-PST-48 Peacock Mtns, AZ	35°21’22.51’’N 113°43’1.47’’W	77, 71±6	15	dense basalt from fluvial sediments	3000	15	16, 17, 18 (9, 11, 11)	6.8-7.3
85-PST-50 Cottonwood Mtns, AZ	35°25’36.76’’N 113°39’51.55’’W	85, 79±6	20	vesicular basalt from fluvial sediments	2500	50	16, 16, 18 (10, 10, 11)	6.4-6.7
86-PST-13 Cottonwood Mtns, AZ	35°13’12.94’’N 113°29’33.52’’W	92, 86±6	5	basalt and granite from fluvial sediments	2700-3000	35	9, 10, 10 (5, 6, 6)	5.0-5.4
86-PST-15 Cottonwood Mtns, AZ	35°13’5.79’’N 113°29’36.43’’W	92, 86±6	5	basalt from fluvial sediments and lavas	3000	50	12, 14, 14 (7, 8, 8)	5.1-5.5
86-PST-22 Peacock Mtns, AZ	35°12’4.47’’N 113°47’32.14’’W	65, 59±6	20	dense basalt	3000	-	9 (5)	5.1
86-PST-26 Aquarius Mtns, AZ	35°5’10.84’’N 113°32’34.09’’W	85, 79±6	20	granite, basalt	2700	60	4, 4, 9 (3, 3, 5)	3.5-4.9
86-PST-46 Cottonwood Mtns, AZ	35°25’29.82’’N 113°38’10.90’’W	87, 81±6	20	dense basalt (from lava flow?)	3000	35	11, 17, 17 (7, 11, 11)	5.8-7.2

Western sector								
PST0016 Ship Mtns, CA	34°32’21.65’’ N 115°21’40.47” W	99, 73±8	6-35	volcanic from alluvial fan	2800	60	9, 10, 10 (5, 6, 6)	4.9-5.4
PST0141 Old Woman Mtns, CA	34°38’2.27” N 115°08’35.43” W	77, 50±8	5-20	volcanic from alluvial fan	2800	100	10, 16, 23 (6, 9, 14)	5.4-8.0
PST0149 Bullion Mtns, CA	34°35’53.57” N 116°10’16.2” W	163, 105.5±7.5	20	granite, gneiss	2700	100	8 (5)	6.0
AM0152 Alvord Mtn, CA	70 m from AM450	190, 138.5±7.5	3.3	granitoid from alluvial fan conglomerate	2700	60	16 (13)	7.4
AM0170 Alvord Mtn, CA	35°06’20.70’’N 116°34’29.88’’W	191, 139.5±7.5	1.1	granitoid from fluvial conglomerate	2700	8	32 (17)	8.5
AM0450 AM0722 Alvord Mtn, CA	35°05’47.52’’N 116°34’10.56’’W	190, 138.5±7.5	3.3	Granitoid from alluvial fan conglomerate	2700	60	28, 34, 45 (20, 18, 29)	8.7-12.4
PST0634 Dagett Ridge, CA	34°48’25.42’’N 116°53’03.24’’W	222, 151.5±7.5	4.5	sedimentary conglomerate, volcanic	2400-2800	60-100	47, 88 (30, 50)	11.5-13.2
PST0695 Newberry Mtns, CA	34°44’09.54’’N 116°43’09.72’’W	208, 140.5±7.5	~30	basaltic andesite from conglomerate	2800	50-200	63, 64, 67, 73, 139 (34, 30, 41, 40, 79)	10.2-20.8
PST1308 Newberry Mtns, CA	200 m from PST0695	208, 140.5±7.5	~30	basaltic andesite	2800	50-150	36, 37, 37, 41, 67 (22, 20, 22, 23, 50)	9.4-14.8

[a] Corrected for regional extension and strike-slip translation.

[b] Up to five largest blocks with lengths A>B>C; mean size $D=\sqrt[3]{(6ABC/\pi)}$ and corresponding short length in brackets.

[c] Calculated from equation 1 with $\xi=1$ and $\rho=875\text{-}1400$ kg/m^3.

Supplementary Table 2. Experiments carried out in this study.

Experiment	Flow front velocity (m s^{-1})	Distance of granular substrate x_1-x_2 (cm) [a]	Rigid substrate	Movie [c]
C1	3.06	0-300		
C2	2.47	0-300		Movie 1
C3	2.55	0-300		Movie 2
C4	1.63	0-300		
C5	2.36	0-300		Movie 3
C6	1.97	0-300		
L1	2.59	82-102	smooth	
L2	2.69	82-102	smooth	
L3	2.82	10-80	smooth	
L4	2.56	10-60	smooth	Movie 4
L5	2.73	10-60	rough 0.7 mm	
L6	2.67	10-60	rough 1.5 mm	Movie 5
L7	2.53	10-60 [b]	rough 0.7 mm	Movie 6
L8	2.47	10-60 [b]	rough 1.5 mm	

[a] See Fig. 5 in paper.
[b] Substrate of 1.5 mm glass beads.
[c] See Supplementary Movies.

Supplementary Discussion

a) Map of Kane Wash area Newberry Mountains, California

The Peach Spring Tuff (PST) exposed in Kane Wash, Newberry Mountains, California, contains locally derived lithic clasts as much as ~140 cm long (Supplementary Table 1). The Kane Wash exposure is approximately 208 km from the Silver Creek caldera of ref. 1 (Fig. 1). Because this area is west of the Colorado River Extensional Corridor (CREC) and is in the Eastern California Shear Zone (ECSZ), the original distance after removing extension and strike-slip translation is probably 133 to 148 km west-southwest of the caldera (Supplementary Table 1). The Kane Wash area is particularly important in our study for constraining the transport distance of the substrate-derived blocks by the PST parent current as well as the flow speed.

In the Kane Wash area, geologic mapping by ref. 2 focused on the facies and distributions of lithic clasts in the Peach Spring Tuff; however, mapping by Brett Cox (USGS, unpublished data) provides important constraints on the derivation of the locally derived, large, basaltic andesite, lithic clasts at location PST0695 and PST1308 (Fig. 3). The following descriptions are based primarily on those by Cox (unpublished) and are augmented by data from refs. 2 and 3 and descriptions from recent field work by us. The Kane Spring paleovalley consists of a westward-draining valley in which a variety of tilted volcanic rocks formed highlands north of the paleovalley and Mesozoic plutonic and metavolcanic rocks formed highlands to the south. Near location PST0695, the PST is deposited on several superposed basaltic andesite lava flows, and locally the upper part contains erosionally resistant boulder flow-breccia (unit Tmb4 in Fig. 3). Rocks in unit Tmb4 form many of the ridges in the southern Newberry Mountains and some of the highlands north of the Kane Spring paleovalley[4]. The northern side of the axial drainage of Kane Springs paleovalley consists of cobble-pebble conglomerate and pebbly sandstone that have bedforms indicating west-southwest directed flow (unit Tkc in Fig. 3). This unit is divided into unit Tkc2 where it is younger than the PST and Tkc1 where it

is older than the PST. The northern flanks of the Kane Spring paleovalley consists of coarse-grained breccia-conglomerate with basaltic andesite and other volcanic rocks as clasts, and minor amounts of interbedded ash tuff, lapilli tuff, and tuffaceous sandstone (unit Tkb in Fig. 3). Locally in unit Tkb, there are very coarse-grained conglomerate beds with only basaltic andesite clasts, and many of these clasts are 1-1.2 m in diameter, possibly 1.5-1.8 m, but blocks are out of the matrix conglomerate (identified in Fig. 3 by unit Tkbc, see also Fig. 4a). Most of unit Tkb is younger than the PST, but locally some is older than the PST.

The PST exposed in Kane Wash is up to 30 m thick, and contains several examples of locally derived lithic clasts that were incorporated into and redistributed within independently moving, relatively small pyroclastic flows or within a single, main pyroclastic flow to form lithic-rich horizons. One type of horizons resulted from small flows and has a maximum lithic clast size of 43 cm, taken as the average long axis of the five largest clasts in a 20x200 cm area[2]. Here we report for the first time another concentration of large, locally derived lithic clasts that were incorporated into the main PST pyroclastic flow, and did not form independently moving pyroclastic flows (location PST0695, Figs. 1b and 3 and Supplementary Fig. 1). These large clasts appear to have been transported independently toward the west-southwest (S65-75W) in the main valley-filling pyroclastic flow along the Kane Spring paleovalley.

There are two possible source areas for the large, basaltic andesite lithic clasts at location PST0695: (1) a nearby basaltic-andesite lava flow (in Tmb4), or (2) a conglomerate with clasts derived from similar basaltic-andesite lava flows (in Tkbc). The nearby basaltic-andesite lava flow forms most of the rocks upon which the PST at location PST0695 was deposited, and flows are exposed within 15 to 20 m behind the location of the photograph in Supplementary Fig. 1. If these basaltic andesite lava flows are the source of large clasts, the distance clasts were transported is probably 20 to 60 m, and longer distances are possible. One important observation that might limit the possibility that the lava flow and boulder flow-breccia are a source for the clasts is that many of the blocks of lava flow on the in situ flows are angular to subangular whereas clasts in the PST are subangular to subrounded. Alternatively, and based on the subrounded shape of many large clasts and the suggestion by Brett Cox (written commun.), the large clasts may have been derived from one of the monomictic (basaltic andesite) boulder conglomerates exposed just below the PST. The nearest boulder conglomerate is 785 m east-northeast of location PST0695 (Tkbc in Fig. 3). To the east-northeast of location PST0695, the substrate is eroded and the nearest exposure is about 175 m at PST1308. At PST1308, the substrate to the PST is sandstone, and there is no evidence of the boulder conglomerate. The map pattern of the boulder conglomerate (Tkbc) is consistent with it being deposited from a small, laterally restricted tributary fan that did not prograde far into the axial drainage. These relations indicate that the large, subrounded basaltic andesite clasts were transported by the valley-filling, PST pyroclastic density current for ~500 m for clasts at PST1308, and ~800 m for clasts at PST0695.

b) Detailed description of key outcrops

Kane Wash exposures (western sector)

Our new field work at location PST0695 shows that the lowest 2 m of the PST contains numerous large, subrounded basaltic-andesite lithic clasts, and one clast is 70x120x150 cm (with the 70 cm length nearly vertical, and partially buried) (Fig. 1b and Supplementary Fig. 1). The mean size of the five largest lithic clasts (except the outlier mentioned above) reported in Supplementary Table 1 is 67±5 cm. The bottoms of the large clasts are about 50 cm above the base of the PST and appear to be in the lithic-rich horizon H1 as defined by ref. 2. Many of the large clasts have an imbricate relation to the basal contact (whether it is clast on clast or

clasts isolated in the finer-grained matrix); the short length is slightly off from vertical and the middle and long lengths form a plane that is shallowly inclined to the basal contact. Additionally, some clasts have the long axis plunging along an azimuth of about 0° to 30°. The moderately developed imbrication and alignment of long axes of clasts are consistent with interaction with and re-alignment by the cross-flowing (S0-30W) internal, lithic-rich pyroclastic flow[2]. At location PST1308, there is one large (>50x70 cm) basaltic andesite lithic clast enclosed by the ash-rich ignimbrite, and two to three complexly interstratified lithic-rich horizons (Supplementary Fig. 2). Large clasts (>10-20 cm) are 0.5-1.5 m above the base of the PST and most of them form the lithic-rich horizon H1 of ref. 2. The bottom of the largest clast is about 1 m above the base of the PST.

The most likely source area of the substrate-derived blocks found at PST0695 and PST1308, the boulder conglomerate Tkbc in Fig. 3, is shown in Fig. 4a. Measurements of 22 subrounded blocks reveal a mean size of 86±18 cm, with the five largest blocks having a size of 111±9 cm. Assuming that the PST parent flows entrained boulders located at top of the conglomerate, we consider in particular blocks L1, L3, and L16-22 in Fig. 4a and whose mean diameter is 91±19 cm, with the five largest blocks having a size of 105±9 cm. Interestingly, these blocks are just larger than the largest blocks at PST0695 and PST1308 (with exception of the outlier 80x120x150 cm at PST0695, Fig. 4), which suggests that our calculated flow velocity up to ~20 m s^{-1} at PST0695 is realistic.

West Gem exposure (western sector)

The Peach Spring Tuff exposed in West Gem area (PST0634) on the northeast flank of Daggett Ridge, California, contains locally derived volcanic lithic clasts as much as 50 cm long and a 120 cm long clast of sedimentary conglomerate (Supplementary Table 1). The West Gem exposure is approximately 222 km from the Silver Creek caldera of ref. 1 (Fig. 1). Because the West Gem area is west of the Colorado River Extensional Corridor (CREC) and is in the Eastern California Shear Zone (ECSZ), the original distance after removing extension and strike-slip translation is probably 144 to 159 km west-southwest of the caldera (Supplementary Table 1).

In the West Gem exposure, the PST is 4.5-m thick with a nonwelded and vitric lower part, is mostly partially welded and incipiently crystallized, and has a nonwelded and incipiently crystallized top[5]. The ignimbrite was deposited on medial to distal alluvial fan sandstone and conglomerate to fluvial sandstone. Most clasts in the sandstone and conglomerate are crystallized volcanic rocks, but plutonic and metamorphic clasts also occur. There is a basal lithic-rich, typically clast supported (although locally matrix supported) layer that contains lithic clasts up to 24 cm in length (Supplementary Fig. 3a) and that we call basal ground layer (BGL) hereafter. Clasts in this BGL are crystallized volcanic rocks that are similar to those in the substrate. The BGL varies in thickness from 50 to 100 cm, and typically is about 60 cm. The variation in thickness results from asymmetric lithic-rich crests that form where the BGL gradationally thickens to form a relief of about 40 cm, and locally as much as 65 cm (Supplementary Fig. 3a). Similar asymmetric crests also occur in the lithic-rich horizons in the PST exposed in Kane Wash.

Isolated, large lithic clasts occur locally in the lower part of the ignimbrite, but with no direct connection to the lithic-rich basal layer. Such a lithic clast shown in Supplementary Fig. 3a (near green notebook) is 30x36x(>)50 cm (the long axis is buried and projects out of the exposure) and is about 50 cm above the top of the BGL. In contrast, other large clasts appear connected to the basal layer. Supplementary Fig. 3a shows a rip-up flap that merges with the basal layer and whose upper part is formed by a lithic-rich, (>)50x60x120 cm clast of sedimentary conglomerate. The sedimentary clast includes volcanic rock fragments similar to the lithic clasts in the BGL; however, it also includes sedimentary rock fragments and has a

more lithic-clastic matrix compared to the tuffaceous matrix in the BGL. So, this sedimentary clast might have been ripped up from the type of material that also provided the clasts for the BGL. How far the lithic-laden boundary flowed is not known.

Alvord Mountain area exposures (western sector)

The Peach Spring Tuff exposed on the east limb of the Spanish Canyon anticline in the Alvord Mountain area, California, contains locally derived lithic clasts as much as 56 cm long (mean size of 45 cm, Supplementary Table 1). The PST exposures are approximately 190 km from the Silver Creek caldera of ref. 1 (Fig. 1). Because this area is west of the Colorado River Extensional Corridor (CREC) and is in the Eastern California Shear Zone (ECSZ), the original distance after removing extension and strike-slip translation is probably 131 to 146 km west-southwest of the caldera (Supplementary Table 1).

The PST is in the Spanish Canyon Formation and can be discontinuously traced for 9 to 10 km around the northward plunging Spanish Canyon anticline[6-8]. The ignimbrite is about 1 to 7-m thick and is mostly nonwelded. Locally, the PST was deposited on basaltic andesite lava flows, or on fine-grained sandstone and mudstone that might represent lacustrine deposits. However, in most areas, the PST was deposited on medium to coarse-grained, arkosic to tuffaceous plutoniclastic sandstones and conglomerates that were probably medial to distal alluvial fan or fluvial deposits. The plutonic and metamorphic rocks exposed in nearby mountains were the source areas for clasts in the sandstone and conglomerate[9-10]. Substrate entrainment by the PST parent flows is also evidenced by characteristics of thin sections that show rock fragments that are consistent with being derived from plutonic and metamorphic rocks exposed in the nearby mountains, or from the substrate alluvial fan sandstone and conglomerate that was strewn with loose, erodible clasts.

A lithic-bearing, basal ground layer (BGL) deposit occurs at locations where the ignimbrite base is exposed; however, the amount of development varies in thicknesses, grain sizes, textures, and structures[7-8]. In exposures on the eastern limb of the anticline where the ignimbrite is 3.3 m thick, the BGL is about 60 cm thick, and it is composed of 5-25% volcanic and granitoid lithic clasts that are 2 to 76 mm in maximum length (for example, locations AM0450, AM0722, and AM0152 in Supplementary Table 1 and Supplementary Figs. 3b-d). Locally, lithic clasts up to 56 cm-long are on the top of the BGL (Supplementary Figs. 3b-c). The matrix between these large clasts is mostly ash and pumice clasts with 2-5% lithic clasts that are less than 5 mm long and is the same as in the surrounding and superjacent main ignimbrite. About 1.1 km northwest of location AM0450, the ignimbrite is about 1 m thick, and the BGL is 10 to 30 cm thick with maximum lithic clasts up to 40 cm in length (AM0170 in Supplementary Table 1 and Supplementary Figs. 3d).

Valentine area exposure (85-PST-50, eastern sector).

The eastern sector of the PST extends from the source area, across a transition zone characterized mainly by normal faulting, and onto the Colorado Plateau. The Colorado Plateau in this area has relatively little faulting. Several outcrops occur in the vicinity of the town of Valentine; in this area the pyroclastic density currents that formed the Peach Spring Tuff flowed across an area of low, rolling hills eroded into Precambrian granitic basement rocks, variably covered by Cenozoic basaltic lavas and scoria. Low profile valleys contained fluvial sediments with gravel to boulder sized clasts (blocks) of granite and basalt, which were the likely sources of substrate blocks entrained by the pyroclastic flows.

In the Valentine area the PST ponded in the broad drainages, filling in the topography and forming a relatively flat upper surface. This surface has been subsequently incised by erosion, exposing cross sections of the tuff. In many locations the deposit overlies early ash- and fine lapilli-beds that record initial phases of the Peach Spring Tuff eruption[11-12]. The main

ignimbrite is massive and poorly sorted, with an inverse coarse-tail graded base evidenced by lapilli and blocks of substrate material (cf. Fig. 1c in paper). The main deposit is relatively structureless (massive), with local upward increases in the size of low-density pumice clasts. The ignimbrite is moderately welded in most of the preserved deposits, which tend to correspond also to deposits that were originally thickest. Vapor phase alteration affects the upper part of the ignimbrite. Both the welding and vapor phase alteration are indicators of relatively hot emplacement temperatures (several hundred degrees C).

c) Velocity of dilute turbulent current required for substrate block entrainment

Here we examine the flow velocity required for entrainment of substrate blocks if the parent PST currents were fully dilute turbulent mixtures. The method is that proposed by ref. 13, which consists of calculating the critical velocity at which blocks entrainment by a hydraulically rough, dilute turbulent current is controlled by a Shield criterion[14]. The assumed bulk density of the current is 2 kg m^{-3} (larger than that of the ambient atmosphere) to 10 kg m^{-3}, which corresponds to a particle volume fraction of ~0.1–1%, as commonly considered in literature (e.g. ref. 15 and references therein). The velocity profile of such a current is

$$U_{(z)} = \frac{u_*}{\kappa} \ln\left(\frac{z}{z_0}\right), \qquad (1)$$

where $U_{(z)}$ is the mean current velocity at height z above the ground, u_* is the shear velocity, κ=0.4 is the von Karman constant, and z_0 is the hydrodynamic roughness. The shear velocity $u_*=\sqrt{(\tau/\rho)}$, with ρ the current bulk density and $\tau=\theta(\rho_p-\rho)gD$ the shear stress generated by the current on the blocks, with θ the Shields number, ρ_p and D the substrate particle density and mean size, respectively, and g the gravitational acceleration. For calculations $z_0=D/30$ is typical of hydraulically rough currents and θ=0.052-0.086 are critical Shield number values for turbulent currents at particle Reynolds number >10^4 (ref .16).

Supplementary Fig. 4 shows the current velocity profile required to entrain the largest substrate-derived blocks found in the PST (Supplementary Table 1 and Fig. 2a in paper). Calculations are made for typical block size D=80 cm and density ρ_p=2800 kg/m^3 and for current height up to 500 m representative of large-volume dilute pyroclastic currents[17-18]. The results show that a velocity of ~300-700 m s^{-1} at height of a few hundred meters above the ground is necessary to entrain the largest PST blocks, which is unrealistic; for instance, maximum current speeds of ~170 m s^{-1} have been reported for the lateral blast eruption at Mount St Helens, 1980, below 4-6 km from vent before the blast rapidly transforms into a density current of speed less than 100 m s^{-1} (ref .17).

Supplementary References

1. Ferguson, C. A., McIntosh, W. C. & Miller, C. F. Silver Creek caldera - The tectonically dismembered source of the Peach Spring Tuff. *Geology* **41**, 3-6 (2013).
2. Buesch, D. C. Incorporation and redistribution of locally derived lithic fragments within a pyroclastic flow. *Geol. Soc.Am.Bull.* **104**, 1178-1193 (1992).
3. Buesch, D. C. Changes in Depositional Environments Resulting From the Emplacement of Large-Volume Ignimbrite, in *Sedimentation in Volcanic Terranes* (Smith, G.A. & Fisher, R.V. ed.), SEPM Special Publication 45: 139-153 (Society for Sedimentary Geology, 1991).
4. Hillhouse, J. W., Wells, R. E. & Cox, B. F. Paleomagnetism of Miocene volcanic rocks in the Newberry Mountains, California: vertical-axis rotation and a polarity transition, in

Overboard in the Mojave (Reynolds, R. E., & Miller, D. M., eds.), 177-195 (California State University Desert Studies Consortium, 2010b).

5. Buesch, D. C. Flow direction indicators in lithic-rich, basal ground layers in western exposures of the Miocene Peach Spring Tuff, California. Presented at American Geophysical Union, Fall Meeting, December 3-7, 2012a, http://agu-fm12.abstractcentral.com.
6. Buesch, D. C. Interstratified arkosic and volcanic rocks of the Miocene Spanish Canyon Formation, Alvord Mountain area, California - Descriptions and interpretations, in *Not a Drop Left to Drink* (R. E. Reynolds, ed.), 190-203 (California State University Desert Studies Center, 2014).
7. Buesch, D. C. Flow direction indicators in lithic-rich, basal ground layers in western exposures of the Miocene Peach Spring Tuff, California. Presented at American Geophysical Union, Fall Meeting, December 3-7, 2012a, http://agu-fm12.abstractcentral.com.
8. Buesch, D. C. Stratigraphy of the possible Peach Spring Tuff in the Alvord Mountain area, California, in *Searching for the Pliocene: Southern Exposures* (Reynolds, R.E., ed.) 179-180 (California State University Desert Studies Consortium, 2012b).
9. Byers, F. M. Geology of the Alvord Mountain Quadrangle, San Bernardino County, California. *U.S. Geol. Surv. Bull.* **1089-A**, p. 196071.
10. Fillmore, R.P. Sedimentation and extensional basin evolution in a Miocene metamorphic core complex setting, Alvord Mountain, central Mojave Desert, California, USA. *Sedimentology*, **40**: 121-142 (1993).
11. Roche, O. *et al.* Dynamic pore-pressure variations induce substrate erosion by pyroclastic flows. *Geology* **41**, 1107-1110 (2013).
12. Valentine, G. A., Buesch, D. C. & Fisher, R. V. Basal layered deposits of the Peach Springs Tuff, northwestern Arizona, USA. *Bull. Volcanol.* **51**, 395-414 (1989).
13. Roche, O. Nature and velocity of pyroclastic density currents inferred from models of entrainment of substrate lithic clasts. *Earth Planet. Sci. Lett.* **418**:115-125 (2015).
14. García, M. Sedimentation and erosion hydraulics, in *Hydraulic Design Handbook* (Mays, L.W., ed.), chap. 6 (McGraw-Hill Professional, 1999).
15. Brand, B.D. & Clarke, A.B. An unusually energetic basaltic phreatomagmatic eruption: using deposit characteristics to constrain dilute pyroclastic density current dynamics. *J. Volcanol. Geotherm. Res.* **243–244**: 81–90 (2012).
16. Buffington, J. M. & Montgomery, D. R. A systematic analysis of eight decades of incipient motion studies, with special reference to gravel-bedded rivers. *Water Res. Res.* **33**, 1993-2029 (1997).
17. Esposti Ongaro, T., Clarke, A. B., Voight, B., Neri, A. & Widiwijayanti, C. Multiphase flow dynamics of pyroclastic density currents during the May 18, 1980 lateral blast of Mount St. Helens. *J. Geophys. Res.* **117**, B06208 (2012).
18. Komorowski, J.-C., Jenkins, S., Baxter, P.J., Picquout, A., Lavigne, F., Charbonnier, S., Gertisser, R., Preece, K., Cholik, N., Budi-Santoso, A. & Surono. Paroxysmal dome explosion during the Merapi 2010 eruption: processes and facies re-lationships of associated high-energy pyroclastic density currents. *J. Volcanol. Geotherm. Res.* **261**, 260–294 (2013).
